# The Syntactic and Semantic Processing of Mass and Count Nouns: An ERP Study

**DOI:** 10.1371/journal.pone.0025885

**Published:** 2011-10-05

**Authors:** Valentina Chiarelli, Radouane El Yagoubi, Sara Mondini, Patrizia Bisiacchi, Carlo Semenza

**Affiliations:** 1 Department of Psychology, University of Trieste, Trieste, Italy; 2 Laboratoire CLLE-LTC (CNRS , UMR 5263), Université de Toulouse 2, Toulouse, France; 3 Department of General Psychology, University of Padova, Padova, Italy; 4 Department of Neuroscience, University of Padova, Padova, Italy; 5 IRCCS Ospedale S. Camillo, Lido di Venezia, Italy; 6 SOC di Neurologia, Ospedale Civile, Gorizia, Italy; Cuban Neuroscience Center, Cuba

## Abstract

The present study addressed the question of whether count and mass nouns are differentially processed in the brain. In two different ERP (Event-Related Potentials) tasks we explored the semantic and syntactic levels of such distinction. Mass and count nouns typically differ in concreteness, hence the effect of this important variable was factorially examined in each task. Thus the stimuli presented were: count concrete, count abstract, mass concrete or mass abstract. The first experiment (concrete/abstract semantic judgment task) involved the interaction between the N400 concreteness effect and the Mass/Count condition, revealing a substantial effect between mass and count nouns at the semantic level. The second experiment (sentence syntactic violation task) showed a Mass/Count distinction on left anterior negativity (LAN) and on P600 components, confirming the difference at the syntactic level. This study suggests that the brain differentiates between count and mass nouns not only at the syntactic level but also at the semantic level. Implications for our understanding of the brain mechanisms underlying the Mass/Count distinction are discussed.

## Introduction

In several languages, *count* and *mass* nouns reflect a basic distinction in our knowledge at the lexical level. This distinction, found already in pre-linguistic infants [1], sits apart within the concrete realm, compact, enduring things (objects) and the stuff (substance) of which they are constituted. *Count* nouns (e.g. dog, chair, knife) apply to perceptual entities that in combination do not yield another entity of the same kind [2]. The samples to which *mass* nouns (e.g. water, sugar, wine) apply are taken as constituting a combination of other samples. The structure of substances, designated by mass nouns, is arbitrary, whereas the structure of objects, designated by count nouns, is not arbitrary [3].

These distinctions are not without problems, however [4]. An exhaustive discussion of the mass count distinction exceeds the aims of this study. Our primary aim is to establish whether differences in brain processing are detectable that may be attributed to a distinction between mass and count. The fundamental ontological distinction between ‘stuff’ or ‘substance’ and ‘objects’ or ‘things’ cannot fully account for the mass/count distinction. Importantly, in fact, count and mass nouns not only identify entities within the concrete domain but also identify different types of abstract concepts. An *idea* is linguistically a count noun: one can have many ideas. *Courage*, on the other hand, is a single unit but linguistically a mass noun. In addition to the mass count distinction, this is the first study to explore the effects of concreteness in the processing of mass and count nouns in the brain.

Differences between the processing of mass and count nouns are not however limited to semantic tasks. Recently, Rothstein [4], has shown that the mass/count distinction is an independent grammatical distinction. In languages like English, French, and Italian, syntactic properties distinguish the two lexical categories of mass and count nouns [5]. Cardinal numerals and quasi-cardinal numerals (e.g. ‘several’) modify count nouns, never mass nouns. Moreover, quantifiers like ‘little’ or ‘much’ modify mass nouns but not count nouns, whereas ‘few’ and ‘many’ modify count nouns but not mass nouns. Count nouns admit a morphological contrast between singular and plural; mass nouns do not, being almost always singular. The pronoun ‘one’ may have a count noun as its antecedent, but not a mass noun. Mass nouns with singular morphology do not tolerate the indefinite article, whereas singular count nouns do. Finally, mass nouns occur only with the plural form of those quantifiers whose singular and plural forms differ. Not all of the properties that distinguish mass and count nouns are, however, found in all languages [6].

The fact must again be stressed that what seems to be an intuitively neat distinction is not at all free from ambiguity [4], [7]–[10]. The instances of uncertainty are potentially endless. In fact in many cases a given word belonging to one or other category is determined by the context. Thus Bunt [9] writes ‘the count-mass distinction is not really a distinction between words, but a distinction between ways of using the words’. For example, a word like ‘stone’ may indicate an individual entity or refer to several things. Likewise one may ‘cook a chicken’, where ‘chicken’ is count, whereas one may ‘cook some chicken’, where ‘chicken’ is mass. Morphosyntactic and semantic properties may be language-specific. ‘Hair’ is mass in English, whereas the French *cheveux* and the Italian *capelli* behave as count. In English such nouns as ‘remains’ or ‘left-overs’ are mass nouns that exist in the plural form. In Italian it is common practice, and grammatically perfectly acceptable, to order ‘a coffee’, meaning ‘a cup of coffee’: the phrase ‘a cup of’ has been deleted from the surface structure. A way around these difficulties is thus to observe that in such cases there is transfer of meaning, e.g. in a figurative sense, as in *smooth waters*. The ultimate answer as to whether a given noun is a count or a mass noun thus depends upon being able to decide for any specific use or alternatively any specific sense. As Barner and Snedeker [11] elegantly showed, the hypothesis that there exist one-to-one mappings between mass-count syntax and semantics is not supported by empirical findings. Participants in their experiments, in fact, based quantity judgments on number when the terms were used with count syntax, but on total amount of stuff when used with mass syntax.

Further problems come from the existence of ‘collective’ mass terms, like ‘furniture’ or ‘silverware’, that show intermediate properties. These terms in fact refer to countable individual entities within the collection while they show the morphosyntactic patterning of mass nouns. Morphosyntax and meaning thus may dissociate in these cases.

Despite all of the ambiguities in the Mass/Count distinction, developmental and psycholinguistic research has highlighted some processing differences (at least in studies based on English). In the infant’s mind the representation of cohesive objects designated by count nouns seems to enjoy a privileged status [1], [12]. Perceptual properties rather than morphosyntactic properties do indeed guide young children’s assignment of newly acquired words to either the mass or the count category before the age of about thirty months. [13].

From a psycholinguistic perspective, research on the Mass/Count distinction was aimed at a better understanding of which category required more cognitive resources to be correctly processed. Gillon, Kehaya & Taler [14] in two on-line lexical decision experiments (simple and morphosyntactic prime) showed that the lexical feature ‘mass’ was computed in both experiments. More recently, Mondini, Kehaya, Gillon, Arcara & Jarema [15] conducted two experiments, a simple lexical decision task with words presented in isolation and a sentence priming task, in which words were primed by a sentential context. In the first experiment, they found similar results to those of Gillon et al. [14] with the mass nouns processed more slowly than the count nouns; however, when a sentential context primed the target word this difference disappeared, suggesting that the relative linguistic ‘complexity’ of the mass nouns with respect to the count ones could be reduced when words were embedded in a semantic and syntactic context.

The present study addresses the question of whether count and mass nouns are differentially processed in the brain. A categorical organization of noun processing has indeed been evidenced in the brain in many studies on both brain-damaged and normal participants [16], [17]. An important and only partly answered question in these studies concerns what determines word-class specific brain location and activity, in particular with respect to the distinction between semantic and lexico-syntactic factors [17], [18]. Information about a grammatical category may be represented independently of its meaning at the level of word form and morphological computation. An ongoing debate in the literature deals with whether the information underlying the differences between mass and count nouns is semantic or syntactic in nature [19].

Neuropsychological investigations of the Mass/Count distinction conducted so far are recent and relatively few in number. Significant findings concern morphosyntactic, conceptual, semantic and lexical aspects, and anatomo-physiological studies are even rarer.

The most convincing findings concern morphosyntactic aspects. Grossman, Mickanin, Onishi & Hughes [20] showed that early dementia patients are particularly sensitive to subtle syntactic distinctions such as those mentioned for mass and count nouns. Shapiro, Zurif, Carey & Grossman [21] showed that agrammatic patients had trouble in discriminating mass and count nouns whose distinction is mainly based at the morphosyntactic rather than at the semantic level. Other investigations with different groups of patients with neurodegenerative pathologies revealed a more lexical-semantic or a more lexical-syntactic deficit in processing either mass or count nouns depending on each specific group [22]–[25].

In the first extensive single case report addressing this issue, a patient was described whose grammar was otherwise perfect but who showed an isolated deficit in the use of the grammatical properties of mass nouns across a series of tasks, as a consequence of focal brain damage [26]. Mondini, Jarema and Liguori [27] have reported the reverse pattern: their patient exhibited a general syntactic deficit whereas his performance was flawless in Mass/Count syntactic tasks. Another interesting finding has been reported by Vigliocco, Vinson, Martin and Garrett [28]: an anomic patient was able to apply proper Mass/Count lexical-syntactic rules to words which she cannot retrieve.

At a general conceptual level, entities named with mass nouns were found [29] to associate with living entities rather than with artifacts within a herpetic encephalitis patient’s memory disorder. Dissociations between mass and count nouns at the semantic and lexical level have been difficult to find. Indeed, past studies on lexical retrieval suggest that mass and count nouns may be supported by largely overlapping regions. In fact, repeated investigations involving a considerable number of aphasic patients found that only one participant, who had a huge left hemisphere lesion, demonstrated a reliable and stable dissociation (count worse than mass) in naming the two categories [30].

Overall, neuropsychological findings on the present topic may seem somewhat disappointing. Mass and count, however, do account together for most of the known world and they seem to overlap quite extensively in brain space. The lack of a clear double dissociation between the two categories in lesion studies seems to support this hypothesis further. A good way to distinguish these two categories may be to tap the time course of their processing. Therefore, measuring ERPs in unimpaired participants may indeed turn out to be an excellent means of studying the Mass/Count processing in physiological terms. In fact, owing to their excellent temporal resolution, event-related potentials can be used as a powerful tool for correlating underlying neural activity to the various temporally distinct phases of linguistic information processing. One of the most important components related to language processing is the N400, a negative component with maximum amplitude peaking around 400 ms post-stimulus onset. It typically shows a centro-parietal scalp distribution in the visual modality and a more frontal or equipotential distribution in the auditory modality. It is thought to reflect semantic expectancy and integration processes; N400 amplitude is especially large for words that are difficult to anticipate and integrate within a sentence context because they are semantically unexpected or incongruous [31]–[33].

In contrast, syntactic processing is reflected by a left anterior negativity (LAN) and a late parietal positivity (P600). The LAN occurs approximately in the same time window as the semantic N400 effect, but generally has a more anterior and left-lateralized scalp distribution [34]–[38]. It is thought to reflect a first syntactic analysis of the sentence to detect potential errors [36]. The P600, in turn, is a positive component that typically shows a parietal scalp distribution with maximum amplitude peaking between 500 and 900 ms [39]–[41]. The P600 is often considered to reflect more controlled syntactic processes, such as syntactic reanalysis and repair [42] or syntactic integration difficulty [43]. N400, LAN and P600 are typically elicited by different kinds of linguistic violations. It is therefore important to mention that these three components have also been observed for more subtle processing differences, without employing the violation paradigm [44], [45].

To our knowledge, only three ERP studies have been published so far that examine the psychological and neural processes underlying the Mass/Count distinction. First, Steinhauer, Pancheva, Newman, Gennari and Ullman [46] utilized a sentence acceptability paradigm to demonstrate that count (vs. mass) nouns elicit a frontal negativity that is independent of the N400 marker for conceptual-semantic processing but resembles anterior negativities related to grammatical processing and syntactic violations. In a second study, in a single-word semantic categorization task (count vs. mass nouns), Bisiacchi, Mondini, Angrilli, Marinelli and Semenza [47] found an early wave peaking at about 150 ms over left frontal sites during processing of count nouns and a potential spread across hemispheres during processing of mass nouns. More recently, Mondini, Angrilli, Bisiacchi, Spironelli, Marinelli and Semenza [48], found a differentially distributed early negativity for mass and count nouns, peaking at 160 ms, in a lexical decision task.

Mass nouns elicited greater left negativity over frontal locations whereas count nouns were more lateralized in the left occipito-parietal sites. These last results have been interpreted in terms of an activation of the linguistic network for mass nouns, more largely distributed around the left frontal regions, and an activation of the left posterior regions for count nouns owing to the activation of a visual representation needed to integrate the concrete count nouns.

As a novelty, with respect to the few previous studies, our study introduces the important dimension of concreteness. This manipulation has two important objectives: first, to avoid a potential confounding of concreteness with that of Mass/Count features and, second, to test the possible interaction between these two dimensions. In the literature exploring the concreteness effect, it has been shown that concrete words are more quickly recognized [49], better remembered [50] and more resistant to brain damage [51], [52]. Several studies have used ERPs to explore the sources of differences in processing of concrete and abstract words [53]–[58]. Though these studies all used different tasks, they unanimously reported that concrete words elicit a more negative N400 than abstract words. Kounios and Holcomb [54] used two tasks (lexical decision and concreteness judgment tasks) to distinguish which theories in terms of the dual code theory [59], [60] and the context availability theory [61] are more consistent with the electrophysiological activation. The dual code theory assumes two separate semantic systems: one composed of a verbal-based code and the other composed of an image-based code. According to this theory, the processing advantage for concrete words occurs because these words activate both verbal- and image-based codes, whereas abstract words activate only the verbal-based code. On the other hand, the context availability theory posits a single system for accessing the meaning of both abstract and concrete words. According to this theory, concrete words can be put in a semantic context more easily and can therefore activate more semantic information. Results of the lexical decision task showed that pseudowords generated larger N400s than real words [62]–[64]. More related to the ERP effect of concreteness, both tasks showed that concrete words were associated with a more negative N400 than abstract words. Moreover, a significant interaction between word concreteness and scalp distribution was also found, such that the amplitude difference between concrete-and-abstract word ERPs was larger over the right than the left hemisphere. This finding suggests that concrete and abstract words were accessing different cognitive and neural processing structures, which is compatible with the dual coding theory of Paivio [60].

In the present study, two different tasks were aimed at exploring the semantic and syntactic levels in mass and count nouns; furthermore, concreteness was manipulated in each task. The words presented were: count concrete, count abstract, mass concrete or mass abstract. In this way we were able to investigate, first, if mass and count nouns are differentially processed in the brain and, second, to evaluate the impact of the concreteness effect on the mass/count distinction. It is important to underline that this investigation, like the few others conducted so far in neuroscience, focuses on the contrast, in the concrete domain, between the nouns of substances and the nouns of objects or enduring things. Furthermore, collective nouns were excluded from the experimental material. The concreteness dimension was also carefully controlled, avoiding any possible ambiguity. Reaction times (RTs), accuracy and ERPs were used as dependent variables. To establish at which level of processing (i.e. semantic and/or syntactic) these events were taking place the late components of the electrophysiological data (i.e., N400, LAN and P600) were analysed in each task and compared between tasks. In line with prior findings on concreteness judgment tasks, we expected that our semantic task would yield a more negative N400 for concrete words compared with abstract words, with a significant interaction between word concreteness and scalp distribution [54]. Moreover, if the semantic aspect of the Mass/Count distinction is critical, we also expected to see a variation between mass and count nouns and/or an interaction with concreteness effect on the N400 component. Finally, in the morphosyntactic task, if the syntactic aspects play an important role in the Mass/Count distinction, then modulations of the LAN and P600 components should be expected (see [46] for the LAN component).

## Results

### Behavioural data

RTs for correct responses and error rates were determined with a repeated-measures ANOVA, using a design with factors in the semantic task: 2 (Mass/Count) ×2 (Concrete/Abstract); and for the morphosyntactic task: 2 (Well-/Ill-formed sentences) ×2 (Mass/Count) ×2 (Concrete/Abstract). Results are presented in Table 1 and 2.

**Table 1:** Mean reaction times (ms) and percentage of errors in the semantic task averaged for each experimental condition.

	RT		Accuracy	
	Concrete	Abstract	Concrete	Abstract
Count	703	857	2.86	24.28
Mass	738	825	5.24	10.24

**Table 2:** Mean reaction times (ms) and percentage of errors in the morphosyntactic task averaged for each experimental condition.

	RT		Accuracy	
	Concrete	Abstract	Concrete	Abstract
**Well-formed sentences**				
** Count**	800	813	3.33	2.86
** Mass**	834	850	7.85	8.33
**Ill-formed sentences**				
**Count**	908	970	14.52	17.62
**Mass**	945	987	24.76	26.21

In the semantic task, RT analyses revealed a main effect of concreteness, with faster RTs for Concrete than for Abstract nouns [720 vs. 841 ms; *F*(1,13) = 17.28; *p*<.01], and no main effects of Mass/Count were found. Interestingly, the Mass/Count×Concrete/Abstract interaction was significant [*F*(1,13) = 12.47; *p*<.01], with a larger concreteness effect for count [*F*(1,13) = 21.83; *p*<.01] than for mass nouns [*F*(1,13) = 9.30; *p*<.01; 154 vs. 87 ms) and with a mass/count effect for both concrete [*F*(1,13) = 4.92; *p*<.05] and abstract nouns [*F*(1,13) = 5.90; *p*<.05]. Moreover, analyses of error rates showed the same tendency, ruling out potential speed-accuracy trade-offs, since fewer errors were made on concrete than on abstract nouns [4.05% vs. 17.26%; *F*(1,13) = 17.68; *p*<.01], and on mass than on count nouns [7.74% vs. 13.57%; *F*(1,13) = 9.80; *p*<.01]. With regards to the significant Mass/Count×Concrete/Abstract interaction [*F*(1,13) = 24.17; *p*<.01], again there was a larger effect of concreteness for count [*F*(1,13) = 25.28; *p*<.01] than for mass nouns [*F*(1,13) = 3.48; *n.s.*; 21.43% vs. 5.00%] and the mass/count effect was largerfor abstract [*F*(1,13) = 18.33; *p*<.01] than for concrete nouns [*F*(1,13) = 3.22; *n.s.*] In particular, Count-Abstract words appeared to be harder to process.

RT analyses in the morphosyntactic task revealed a main effect of sentences, with faster RTs for well-formed than for Ill-formed sentences [824 vs. 952 ms; *F*(1,13) = 24.46; *p*<.01], a main effect of Mass/Count, with faster RTs for sentences with count nouns [872 vs. 904 ms; *F*(1,13) = 8.33; *p*<.05], and a main effect of Concrete/Abstract, with faster RTs for concrete sentences [872 vs. 905 ms; *F*(1,13) = 6.64; *p*<.05]. In this analysis no interactions were found.

Analyses of error rates revealed similar results to those for RTs. Participants made fewer errors in the well- than in the ill-formed sentences [5.59% vs. 20.77%; *F*(1,13) = 41.24; *p*<.01], in sentences with a count rather than a mass noun [9.58% vs. 16.78%; *F*(1,13) = 34.55; *p*<.01]. Moreover, results showed that the Type of sentence interacts with the Mass/Count effect [*F*(1,13) = 7.27; *p*<.05]. More precisely, post hoc analyses showed that the effect of Mass/Count was significant for the two kinds of sentences but with different levels: [*F*(1,13) = 7.51; *p*<.05] for the well-formed sentences and [*F*(1,13) = 23.62; *p*<.01] for the ill-formed ones.

### ERP data

**Figure 1:**
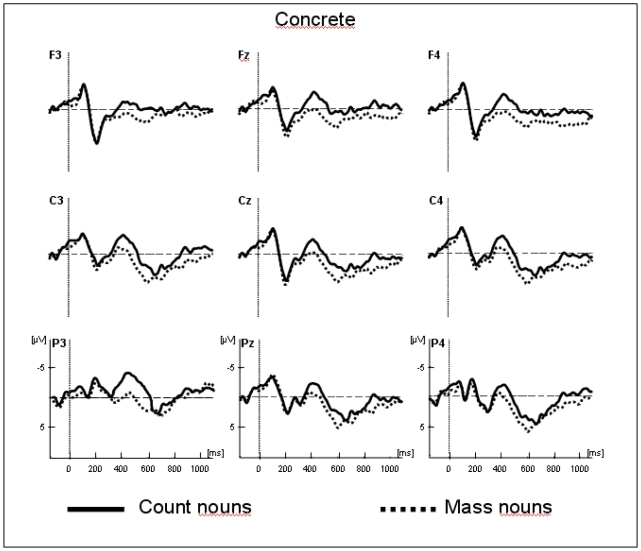
Overlap of the grand average ERPs in the semantic task for concrete count and mass nouns recorded from nine selected scalp sites. As observed in Figure 2a, the brain waves in the mass condition diverge from those in the count condition as early as about 250 ms and particularly in the left parietal site (P3 electrode).

**Figure 2:**
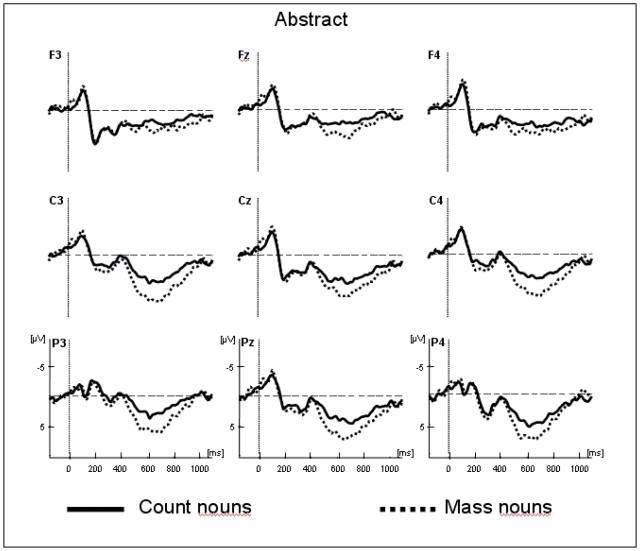
Overlap of the grand average ERPs in the semantic task for abstract count and mass nouns recorded from nine selected scalp sites.

**Figure 3:**
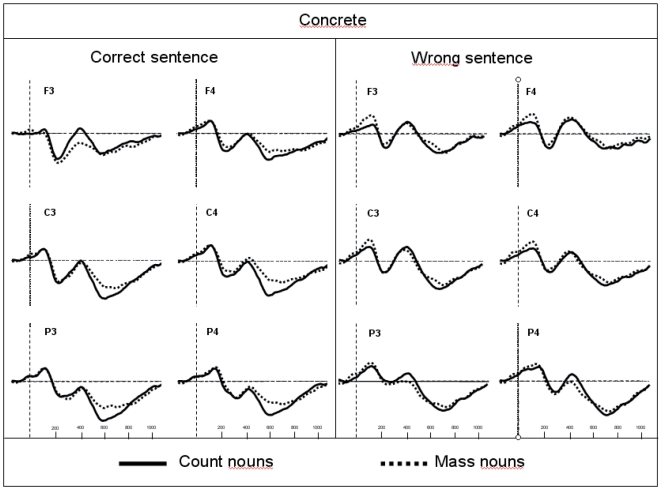
Grand average ERPs recorded in the morphosyntactic task for concrete count and mass nouns recorded from six selected scalp sites. Recordings from well-formed sentences are presented in the left panel and from ill-formed sentences in the right panel.

**Figure 4:**
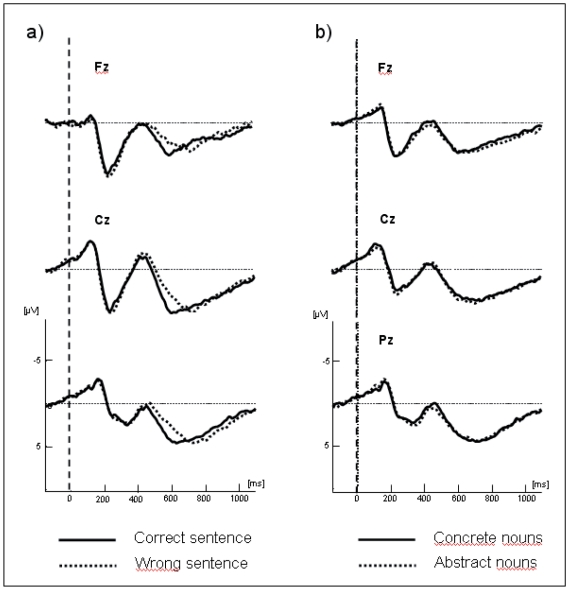
Grand average ERPs recorded in the morphosyntactic task at midline electrodes. In the left column ERPs are compared between (a) well- and ill-formed sentences and (b) between concrete and abstract nouns in the right column.

The traces presented in Figures 1 and 2 show the grand average potentials for the semantic task recorded at nine representative electrodes. The ERPs elicited by concrete count and mass nouns are superimposed on Figure 1, and the ERPs elicited by abstract count and mass nouns are superimposed on Figure 2. As shown in these figures, within the initial 300 ms, count and mass nouns elicited similar N1-P2 complexes whether they were concrete or abstract. Interestingly, only concrete nouns elicited a negative component, starting at 300 ms, which is larger for count than for mass nouns. This effect is very similar to the N400 component reported in previous language studies. With respect to the morphosyntactic task, visual inspection seems to reveal a negative difference only for correct concrete sentences, starting around 150 ms, which is larger for count than for mass nouns and is distributed around the left anterior sites (see Figure 3, left panel). This effect can be related to the LAN component. This first negative peak was followed by a second positive peak, peaking between 500 and 800 ms, which is larger for count than for mass nouns and distributed around the posterior sites (see Figure 4). Moreover, the direct comparison between well-formed and ill-formed sentences seems to reveal a latency difference on the P600 component with well-formed sentences peaking earlier than ill-formed sentences (see Figure 4, left panel).

In order to examine these effects in further detail, three latency ranges of main interest were distinguished, both from visual inspection of the ERP traces and from comparison with previous results available in the literature: the 0–300 ms interval, to test the N1-P2 complexes, the 300–500 ms interval, to test the N400 and the LAN components; and the 500–800 ms interval to test the P600 component.

### Task effects

In order to explore potential task effects for the processing of mass/count nouns, a direct comparison across tasks was performed. To compare the same 120 items in each task and to avoid a potential effect of grammatical violation, only the items corresponding to the well-formed sentences (for the morphosyntactic task) were used in the analysis.

**Figure 5:**
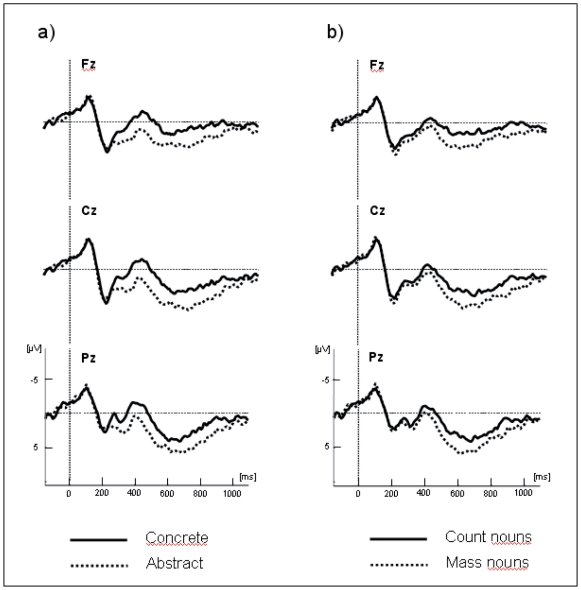
Grand average ERPs recorded in the semantic task at midline electrodes (Fz  =  Frontal; Cz  =  Central, Pz  =  Parietal). In the left column ERPs are compared between (a) concrete and abstract conditions; the brain waves in the abstract condition diverge from those in the concrete condition as early as about 225 ms; (b) mass and count nouns in the right column. In this and subsequent figures, amplitude (µV) is represented on the ordinate, with negative voltage up, and time (ms) on the abscissa.

From zero to 300 ms, there were no significant main effects or interactions at either midline or lateral electrodes. From 300 to 500 ms ANOVAs showed a main effect of concreteness, both for midline [*F*(1,13) = 7.32; *p*<.05] and lateral electrodes [*F*(1,13) = 9.26; *p*<.01]: concrete words elicited larger negativities than abstract words (see Figure 5). Also, at lateral electrodes a significant Concrete/Abstract×Hemisphere interaction was found [*F*(1,13) = 7.12; *p*<.05]. Post hoc analysis revealed that this interaction was due to the concrete words producing a more negative response than the abstract words over the right hemisphere [*F*(1,13) = 11.04; *p*<.01] than over the left hemisphere [*F*(1,13) = 6.46; *p*<.05].

Midline electrodes show an interaction between Mass/Count and Concrete/Abstract [*F*(1,13) = 6.73; *p*<.05; see Figures 1 and 2]. Follow-up analysis demonstrated that the difference between count and mass nouns was only significant for concrete nouns [*F*(1,13) = 6.52; *p*<.05]. Moreover, the interaction between Task, Mass/Count and Concrete/Abstract was also significant [*F*(1,13) = 5.21; *p*<.05]. More precisely, post hoc analysis showed that the difference between concrete count and concrete mass nouns was only significant in the semantic task [*F*(1,13) = 10.24; *p*<.01]. These findings showed that, in the semantic task, the condition eliciting the largest negativity within this temporal window was that of concrete count nouns.

The Mass/Count by Concrete/Abstract interaction was also significant at lateral electrodes [*F*(1,13) = 6.17; *p* = .05]. The difference between count and mass nouns was significant only for concrete nouns [*F*(1,13) = 7.68; *p*<.05]: the concrete count nouns elicited larger negativity than concrete mass nouns. Also, at lateral electrodes a significant Task×Mass/Count×Concrete/Abstract×Hemisphere×Localization interaction was found [*F*(2,26) = 4.12; *p*<.05]. Post hoc analysis revealed that the interaction between Mass/Count by Concrete/Abstract was strongest in the left posterior area [*F*(1,13) = 9.24; *p*<.01] for the semantic task and in the left anterior area for the morphosyntactic task [*F*(1,13) = 5.94; *p*<.05].

From 500 to 800 ms, the concreteness effect was significant only at lateral electrodes [*F*(1,13) = 6.22; *p*<.05]: abstract nouns were more positive-oriented than concrete nouns. In addition, there was a significant Task×Mass/Count interaction both for midline [*F*(1,13) = 4.51; *p*<.05] and lateral electrodes [*F*(1,13) = 3.28; *p*<.05]: mass nouns elicited larger positivities than count nouns in the semantic task and the reverse pattern was observed in the morphosyntactic task (larger positivities for count nouns). Moreover, a Task×Mass/Count×Localization was observed in lateral electrodes [*F*(2,26) = 5.26; *p*<.05]. Follow-up analysis showed that the interaction between Task and Mass/Count was only significant at posterior area [*F*(1,13) = 6.41; *p*<.05].

### Morphosyntactic task

**Figure 6:**
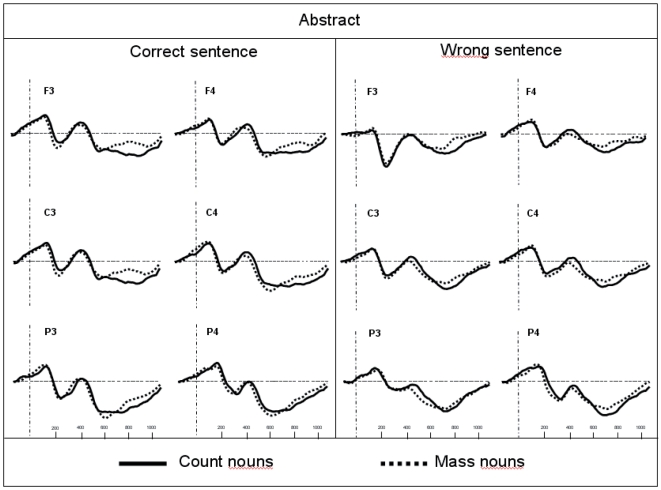
Grand average ERPs recorded in the morphosyntactic task for abstract count and mass nouns recorded from six selected scalp sites. Recordings from well-formed sentences are presented in the left panel and from ill-formed sentences in the right panel.

From zero to 300 ms, ANOVAs showed an interaction between Sentences and Mass/Count at lateral electrodes [*F*(1,13) = 6.11; *p*<.05]. In this case, the difference between count and mass nouns was significant only for ill-formed sentences [*F*(1,13) = 7.24; *p*<.05] and in particular, the mass noun condition elicited larger negativity than count nouns. Moreover, results showed a triple interaction between Sentence, Mass/Count, and Concrete/Abstract [*F*(1,13) = 15.39; *p*<.01]: the difference between count and mass nouns was significant only for the concrete ill-formed sentences [*F*(1,13) = 14.31; *p*<.01]. Also, at lateral electrodes a significant Sentence×Mass/Count×Concrete/Abstract×Localization interaction was found [*F*(2,26) = 4.25; *p*<.05]. Post hoc analysis revealed that the difference between count and mass nouns observed for the concrete ill-formed sentences was strongest in the anterior [*F*(1,13) = 8.71; *p*<.05] and central areas [*F*(1,13) = 6.03; *p*<.05] (Figures 3 and 6).

From 300 to 500 ms, ANOVAs showed a main effect of Sentences, both for midline [*F*(1,13) = 12.52; *p*<.01] and lateral electrodes [*F*(1,13) = 10.21; *p*<.01]: ill-formed sentences elicited larger negativities than well-formed sentences. Also, midline electrodes showed a triple interaction between Sentences×Mass/Count×Electrodes [*F*(3,39) = 2.89; *p*<.05]. The difference between count and mass nouns was localized to Fz [*F*(1,13) = 5.91; *p*<.05]. The frontal distribution of the difference between count and mass nouns is further supported by the interaction between Sentences, Mass/Count, Hemisphere, and Localization [*F*(2,26) = 11.46; *p*<.01]: the difference between count nouns and mass nouns was localized to the left anterior regions [*F*(1,13) = 15.57; *p*<.01] and showed that count nouns elicited a larger negative peak than mass nouns.

In the 500–800 ms latency window, ANOVAs showed a main effect of Mass/Count, both for midline [*F*(1,13) = 13.87; *p*<.01] and lateral electrodes [*F*(1,13) = 11.13; *p*<.01]. Specifically, count nouns elicited larger positivities than mass nouns. Also, midline and lateral electrodes show an interaction between Sentences and Mass/Count, [*F*(1,13) = 4.12; *p*<.05] and [*F*(1,13) = 3.30; *p*<.05] respectively. The difference between count and mass nouns was larger for well-formed sentences. Moreover, the Sentence×Mass/Count×Concrete/Abstract interaction was also significant for lateral electrodes [*F*(1,13) = 16.23; *p*<.01]: the difference between count nouns and mass nouns was larger for concrete and well-formed sentences. Finally, the interaction between Sentences, Mass/Count, Concrete/Abstract and Localization was significant [*F*(2,26) = 14.80; *p*<.01], showing that well-formed sentences containing count nouns elicited a larger positive peak in the central [*F*(1,13) = 6.42; *p*<.05] and posterior areas [*F*(1,13) = 10.90; *p*<.01] compared with well-formed sentences containing mass nouns.

### Partial least square

**Figure 7:**
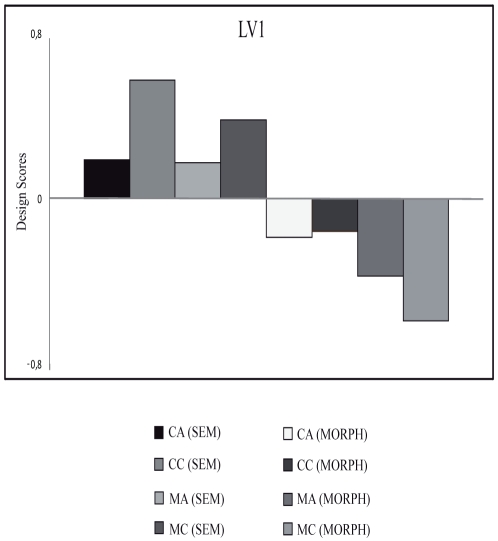
Design scores from PLS analysis for the two tasks (semantic and morphosyntactic). CA: Count Abstract, CC: Count Concrete, MA: Mass Abstract, MC: Mass Concrete, SEM: Semantic Task, MORPH: Morphosyntactic Task. Note that for Morphosyntactic tasks only well-formed sentences were used.

**Figure 8:**
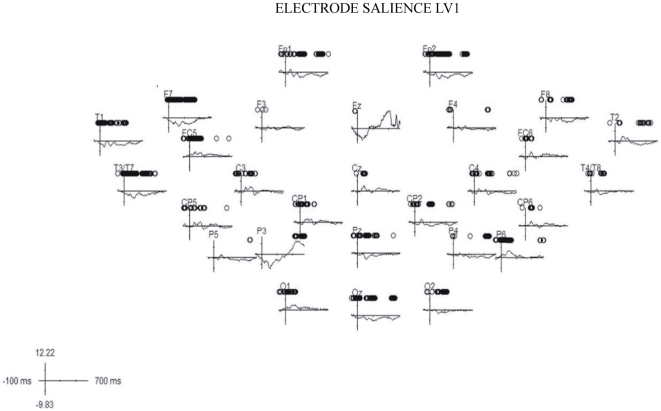
Electrode saliencies relative to the first latent variable of PLS analysis for all recorded electrode sites. Black circles represent the time points with stable effect (*p* < .01).

The PLS analysis included the ERPs elicited by Mass and Count (for both concrete and abstract) trials in both the experiments for the epoch 0–700 ms and all electrodes except the ocular channels. The permutation test revealed only one significant latent variable (LV1; p < 0.001) that accounted for 43.57% of the covariance. LV1 distinguished clearly between the ERPs elicited by the correct responses in the semantic and in the morphosyntactic tasks (Figure 7). The electrode salience (Figure 8) for this latent variable was associated with a sustained modulation over fronto-temporo-parietal and occipital left sites in the time window between 300 and 500 ms, probably reflecting the differences in the cognitive aspects involved in the two different tasks.

## Discussion

Both the behavioural and ERP data highlight some interesting results regarding the distinction between mass and count nouns and their interaction with concreteness. We will first discuss the results obtained in each task and then compare them.

### Semantic categorization

This task revealed that concrete nouns, compared with abstract ones, elicit a larger negativity in 300–500 ms following the onset of stimulus. This negative ERP may exhibit a typical N400 concreteness effect [53]-[55], [57], [58]. Our results also revealed a significant interaction between word concreteness and lateralization, indicating that concrete and abstract words yield significantly different ERPs over the right hemisphere, but not over the left hemisphere. This finding is consistent with the extended dual-coding hypothesis [53], which suggests that both superior associative connections and the use of mental imagery contribute to the processing advantages of concrete words over abstract words. Therefore, the most interesting result obtained in this task involves the interaction between this N400 concreteness effect and the Mass/Count condition. In fact, although there is no significant difference between N400 amplitude for abstract mass and count nouns, a difference is found for concrete nouns (i.e. larger negativity for concrete count nouns relative to concrete mass nouns). Increased negativity for concrete count nouns could be due to the combined activation of two different cognitive processes. Both superior associative connections and easier access to mental imagery would contribute to a processing advantages for concrete count nouns over concrete mass nouns. The process of quantification may explain this result. In fact, the possibility of detecting a structure for a given object could be specifically derived from the Mass/Count distinction, but only for the concrete noun category (e.g. book vs. milk). This concept has been defined ‘structural arbitrariness’ [3], [47] and refers to the fact that whereas substances have arbitrary structures, objects have a structure that is not arbitrary, and are thus more easily identified. As a consequence, when (concrete) count nouns denote real-world objects that have non-arbitrary structures, they are also quantifiable at a numeric level. In contrast, (concrete) mass nouns do not have this property, probably because of their arbitrary structure. Thus, the representation of objects may be constructed through discrete quantification, whereas the representation of substances may be built through quantification along a continuum [3]. This interpretation is also confirmed by the behavioural data (error rates and RTs), which show a slowing down of RTs and an increase in error rates for abstract count nouns compared to concrete count nouns. Furthermore, this interpretation fits with the topographical distribution data showing that building and using representations of concrete count nouns seems to activate the left posterior areas. Although ERP methodologies could not provide a clear topographical resolution as fMRI does, it is interesting to note that the distribution of this effect is close to that described for counting and mathematical calculation in fMRI studies [65]. Thus, it could be interesting in the future to verify the possibility of similar activation between count-concrete nouns and numbers by using fMRI methods. Again, consistently with the present study, Mondini et al. [48] have recently reported for the first time the same particular left posterior localization associated with concrete noun processing. The authors interpreted this topographical pattern which was observed specifically for concrete count nouns as suggesting that this category of nouns needs to be integrated with their corresponding visual representations (mostly stored in posterior middle and inferior temporal gyri, [66]).

Interesting results were also found in the latency window corresponding to the P600 component. It is important to point out that the P600 generally reflects aspects of sentence structure integration, and therefore it can be difficult to relate significant differences in this component range to a task in which only single words are presented. In this case, however, a significant effect was found for the Concrete/Abstract factor, in which abstract nouns elicited a larger (more positive) P600 than concrete nouns. This finding should not be surprising in view of the plausible explanation that, given the instructions for the task, participants could adopt a strategy that helps them to decide whether the word is concrete or abstract by activating a sentence context that situates the word illustratively. Moreover, a significant effect of the Mass/Count factor is present at both the midline and lateral electrodes: mass nouns elicit larger P600 components than count nouns. Given the above-mentioned interpretation, if participants activate a sentence context to decide whether the word is concrete or abstract, it is reasonable to expect that this context could spread activations of certain morphosyntactic characteristics of the Mass/Count distinction at the level of the mental lexicon (e.g. the use of particular quantifiers or articles, whether or not the plural form is used).

Finally, it is also worth mentioning the behavioural data: the reaction times and error rates are significantly greater for abstract count nouns. This finding may show that the participants had trouble in deciding whether or not certain count nouns were abstract. Moreover, this effect supports the interpretation that in order to solve the problem with abstract count nouns participants actually needed to use them in a sentence context.

In conclusion, the most important result underlined by this semantic experiment is that a difference was found between count and mass nouns at the semantic level (N400) if concreteness effect is taken into consideration. The most negative peak in the N400 latency band for count concrete condition could be explained by the combination of two different processes: the first connected with the aspect of mental imagery and the second connected with the aspect of ‘structural arbitrariness’ and, possibly, quantification.

### Morphosyntactic task

The first issue to be addressed is the large discrepancy between the error rates for well- and ill-formed sentences. This effect is mainly owed to the errors on the ill-formed sentences containing mass nouns in general; it is even more pronounced for abstract mass nouns. Although it is certainly very important to investigate the reasons behind such a discrepancy (an issue that will also have bearing on future experiments), the present study concentrated on the phenomena highlighted in the well-formed sentences, since there were not enough items in the ill-formed sentence condition for a valid analysis of the traces to be performed. Thus, this discussion will be focused mainly on analysing the results obtained for the well-formed sentence condition in the two principal latency windows. Another interesting effect related to ill-formed sentences in the morphosyntactic task, however, is the difference between mass and count in ill-formed concrete sentences in the first latency band (0–300 ms; see Figure 6). This strange effect could be due to the quantifier that precedes the final word and it could be related to the expectation of a certain type of final word (i.e., the concept of ‘cloze probability’) [33]. In this specific case, mass syntax (but only for concrete words) seems to be more disturbed than count syntax by a specific violation, probably because it generates a stronger expectation about the final word than count syntax. This fact could, at least in part, explain the larger number of errors in the behavioural data for ill-formed mass than for ill-formed count sentences. The reason for this particular effect of expectation of mass syntax with respect to count syntax is not so clear, however, future research should also concentrate on this aspect of mass/count distinction.

In the latency window between 300 and 500 ms, a significant effect of Mass/Count only in correct sentence condition, particularly localized over left anterior regions, could easily be interpreted as an activation of the LAN component (Left Anterior Negativity). This component has been associated in the literature with a first syntactic processing of words in a sentence [36] and it has also been found in a previous mass/count study [46]. Thus, the localization, because it is confined to the left anterior region (see Figure 3) and has been observed also in Steinhauer et al. [46], where sentence contexts were provided, ruled out the possibility of interpreting this differential wave as an N400 (generally distributed over centro-parietal area) and confirmed the presence of a LAN modulation in morphosyntactic task. According to Steinhauer et al.'s [46] interpretation, the LAN distinction between mass/count nouns relies on a grammatical feature differentiation (e.g. count nouns can take the indefinite article, whereas mass nouns cannot) at the mental lexicon level when a contextual framework is provided. The polarity of this distinction, however, remains unclear. In particular, which is the ‘default value’, is it the ‘mass’ condition that appears in all natural languages or the ‘count’ condition that needs shorter RTs as prompted in (at least) one behavioural study [14]? Looking at our behavioural data in the morphosyntactic task, it could be suggested that count nouns are the default and unmarked case at least for Italian-speaking people [15]. Thus, the negative increase for count condition in ERP data could be owed to the activation of cognitive processes related to the quantification of these words at a linguistic level. In this case, the absence of an interaction with the Concrete/Abstract factor (observed only for semantic task) would be determined by the fact that participants are not analyzing a conceptual aspect directly related to the world of physical objects that these words denote; instead, they are only analyzing the words’ linguistic properties (which happen to be more syntactic in nature). Other research (and in different languages) needs to be conducted to explore which noun category (mass or count) is the default value and thus demands fewer cognitive resources to be processed.

In the latency window between 500 and 800 ms, which is the range of the P600 component, a significant effect of the Mass/Count factor was particularly prominent in the central and posterior regions (see Figure 3). The well-formed sentences containing count nouns elicit the largest positive peak. The P600 component, as previously mentioned, generally occurs in association with sentence contexts and either serves the goal of disambiguating sentences that contain ambiguous syntactic structures or, according to some authors, allows for a final processing of sentence meaning, taking into consideration the characteristics of both its semantic and syntactic structures. The present P600 Mass/Count effect was independent of any violation (well-formed sentences only) and may be more directly linked to a grammatical distinction between sentences that vary in complexity [43] or as a function of the number of alternative syntactic structures that are compatible with the input (syntactic ambiguity) [40], [67]. The P600 distinction, however, also confirms the importance of syntactic differences between mass/count nouns.

### General discussion

The present study is at the forefront of the psycholinguistic and neuropsychological debate on the mass/count distinction, offering some innovative findings. Many researchers have long been seeking to discover if there is a semantic difference between mass and count nouns, in addition to the syntactic difference between the two. Many studies have argued that the syntactic dimension dominates the distinction, and to some extent our experimental data provide evidence to that effect. In fact, the syntactic difference between the two types of nouns emerges clearly not only in the morphosyntactic task, but also during the semantic categorization task (in the second latency window). The present study, however, by adding the dimension of concreteness to the mass/count, distinction has revealed a substantial difference between mass and count nouns at the semantic level. In fact, up to now, only some studies on infant development and a few studies on patients with dementia and herpetic encephalitis have indicated the presence of any relevant semantic aspect. of the presence of an interaction between the two factors in the semantic categorization task, as previously discussed, supports our interpretation that two different phenomena are at play: one principally concerning imageability, and the other (within concrete nouns) concerning the arbitrariness of the structure of countable nouns and the possibility of quantifying them individually. This interaction at the semantic level should thus be kept in mind in future experiments on this topic, regardless of whether they utilize behavioral or brain-imaging methods.

A further relevant aspect has to be kept in mind for future experiments. In fact, while in the morphosyntactic task a short sentence was presented in each trial, in the semantic task only one word was presented. As a result, in the morphosyntactic task, it is possible that some differences between mass and count nouns were masked by a sort of “context effect”. Therefore, it will be important in future experiment to control for this aspect.

Importantly, the PLS analysis provided the opportunity to study the effects of semantic and morphosyntactic tasks in relation to the mass-count and abstract-concrete distinctions that could not clearly emerge from classical ERPs analysis. One latent variable emerged from this analysis that clearly differentiates semantic and morphosyntactic processes (see Figure 7). PLS analysis shows a differential effect of the mass-count and the abstract-concrete distinction in the two tasks. This latent variable is expressed in a different way in the two dimensions. In the morphosyntactic task, mass nouns showed greater design scores than count nouns, suggesting that processing differences between mass and count emerge clearly in this task. Another reason why PLS was useful is that the semantic and the syntactic task built for this investigation differ in some important respect and are thus hard to compare. Crucially, for instance, while a short sentence is presented in the syntactic task, only one word is presented in the semantic task. For the reasons explained in the Methods section, PLS minimizes the effect of such a difference.

In addition to this result, the present experimental investigation opens up a discussion of the issue of what the fundamental basis might be between the two noun typologies. Proponents of the ‘mass nouns as the default’ theory are situated in the framework of cross-linguistic studies and emphasize the existence of these two noun typologies in all languages. Conversely, proponents of the opposing theory base their framework on behavioural results (reaction times slow down for mass nouns in healthy participants and dementia patients tend to lose information on mass nouns first). The data reported here seem to coincide with the latter theory.

### Conclusion

This experimental investigation has turned out to be quite useful for understanding certain linguistic and cognitive mechanisms governing the mass/count distinction, which up to now, remained unclear. Nonetheless, the resolution of the spatial localization afforded by the ERP method is by no means comparable to that which can be obtained with the functional Magnetic Resonance Imaging (fMRI) method. Thus, although the present study provides important information on the time course of the events that unfold during the processing of these noun typologies, it is not yet possible to draw definitive conclusions on the spatial distribution of this cognitive process. An fMRI experiment on this topic is in progress.

## Materials and Methods

### Participants

After informed consent was received from them, 16 Italian students were administered the two tasks in different order. Because of the large number of artefacts, the data from two participants were excluded from the ERP grand averages. Thus, a total of 14 adults (six men and eight women, mean age 24 years; range  =  19–34 years) were tested individually in a session that lasted for about two hours. All were right-handed, neurologically normal (none of the participants were under specific medication), and had normal or corrected-to-normal vision (as controlled for at the beginning of the experiment).

The research was approved by the Ethical Committee of the Department of Psychology of the University of Trieste. Participants signed an informed consent, data were analyzed anonymously and the investigation has been conducted according to the principles expressed in the Declaration of Helsinki.

### Stimuli

Stimuli were chosen with particular care. Four independent judges selected the whole list of nouns in order to avoid all potential confounds mentioned in the introduction. Any ambiguous item in terms of mass/count lexical category or in terms of concreteness/abstractness was ruled out from the list. Thus terms like *caffé*, ‘coffee’, that in different contexts can be commonly used in Italian either as a mass or as a count noun, were excluded from the experimental material. Collective nouns were also excluded. The list comprised 120 Italian nouns (see Appendix S1), subdivided into four different categories: (a) thirty singular concrete count nouns (e.g. *libro*, ‘book’); (b) thirty singular abstract count nouns (e.g. *ipotesi*, ‘hypothesis’); (c) thirty singular concrete mass nouns (e.g. *burro*, ‘butter’); (d) thirty singular abstract mass nouns (e.g. *umiltà*, ‘humility’). Length (number of letters), Frequency, Familiarity, Imageability and Age of Acquisition of nouns was considered. Frequency values were taken by the BDVDB (*Base di Dati sul Vocabolario di Base della Lingua Italiana*, [68]), whereas Familiarity, Imageability and Age of Acquisition were collected through questionnaires. These experimental subjects were balanced with respect to gender, age, and degree of education. Three different questionnaires were administered to 142 Italian native speakers who would not participate in the ERPs experiment. They were preliminarily asked to judge whether a given noun was abstract or concrete. No ambiguous item was found in this respect. The rating scale for Familiarity, Imageability and Age of Acquisition was a seven-point scale. Length, Frequency and Familiarity did not differ for the four classes of nouns [*F*(3,116) < 1; *F*(3,116) < 1; *F*(3,116) = 1.29]. A difference emerged, instead, for Age of Acquisition [*F*(3,116) = 35.14; *p* < .0001], with concrete nouns acquired before abstract nouns [*t(118) = *-8.42; *p* < .001]. Imageability was also significant across categories [*F*(3,116) = 176.21; *p* < .001], highlighting the difference between concrete and abstract nouns [*t*(118) = 20.8; *p* < .001]. This last result is congruent with the expected correlation between Imageability and Concreteness of words [69].

The same 120 stimuli were used for the semantic and the morphosyntactic tasks. No extra stimuli were needed for the semantic task. For the morphosyntactic task, however, nouns were presented at the end of a very short sentence: two words + quantifier + item. The quantifier could be either *un po’ di* (i.e. some), matching the mass noun category, or *un/una* (i.e. a_femm/masc_), matching count nouns. The matching quantifiers and nouns yielded one hundred and twenty well-formed sentences (e.g. *questo è un cavallo*, ‘this is a horse’), and one hundred and twenty syntactically ill- formed sentences (e.g. *questo è un po’ di cavallo*, ‘this is some horse’).

In each experiment stimuli presentation was controlled by E-prime [70]. Nouns were displayed at the centre of a computer screen placed 70 cm in front the participant. All stimuli were displayed in white on a grey background.

### Procedure

In the present study, the main goal was to explore the semantic and syntactic levels of mass and count nouns. To avoid a potential effect of repetition, the administration order of the semantic and morphosyntactic tasks was counterbalanced across subjects. Participants were instructed to respond as quickly and accurately as possible. For each task the set of stimuli was divided into two blocks, with an equal number of experimental conditions within each block. The order of blocks was counterbalanced across participants, and the list of stimuli was randomized for each participant within each block. Each block of trials lasted approximately 6 min, and short rest periods were provided between blocks. To familiarize participants with the task, each task started with a practice session.

**Figure 9:**
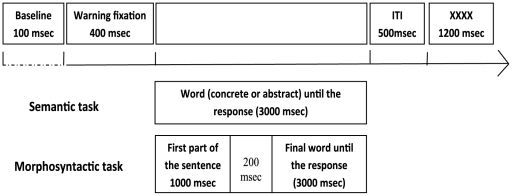
Sequence of events within a trial of each task.

In the semantic task, participants were instructed to press the left (or right) button if the stimulus was a concrete word and the right (or left) button if the stimulus was an abstract word. In the morphosyntactic task, participants were instructed to press the left (or right) button if the sentence was well-formed and the right (or left) button if the sentence was ill-formed. Response hands were counterbalanced across participants. The sequence of events within a typical trial was as follows (see Figure 9): a warning-fixation stimulus was displayed at the centre of the screen for 400 ms followed by the stimulus, which remained until the participant responded (for the morphosyntactic task the stimuli were preceded by the first part of the sentence presented for 1000 ms with a blank screen of 200 ms between the first part of the sentence and the stimuli). A clock began timing when the stimuli appeared and stopped when the participant pressed one of the two response buttons. Participants were given 3000 ms from stimuli onset to give their answers. The ITI lasted for 1700 ms, following the participant’s response. During the ITI, four Xs appeared at the centre of the screen to inform participants that they could blink and move their eyes. Participants were asked to refrain from moving (except for the button press response) and blinking during the critical phase of EEG recording.

### Data acquisition and analyses

EEG was recorded from 28 scalp electrodes mounted on an elastic cap and located at standard left and right hemisphere positions over frontal, central, parietal, occipital and temporal areas (International 10/20 System, at Fz, Cz, Pz, Oz, Fp1, Fp2, F3, F4, C3, C4, P3, P4, P5, P6, O1, O2, F7, F8, T3, T4, T5, T6, Fc5, Fc6, Cp1, Cp2, Cp5 and Cp6). These recording sites plus an electrode placed over the right mastoid were referenced to the left mastoid electrode. The data were recorded continuously throughout each task by a SynAmps amplifier and NeuroScan 4.3 software. Each electrode was re-referenced off-line to the algebraic average of the left and right mastoids. Impedances of these electrodes never exceeded 5kΩ. The horizontal electrooculogram (HEOG) was recorded from a bipolar montage with electrodes placed 1 cm to the left and right of the external canthi; the vertical (VEOG) was recorded from a bipolar montage with electrodes placed beneath and above the right eye, to detect blinks and vertical eye movements. The EEG and EOG were amplified by a SynAmps amplifier with a band pass of 0.01–30 Hz, filtered for 50 Hz and digitized at 500 Hz. EEG epochs containing EOG activity were detected by wavelet analysis and corrected by means of a regression method in the time domain [71]. ERP were extracted by averaging trials separately for subjects, electrodes, and experimental conditions.

ERP data were analysed for correct responses only by computing the mean amplitude in selected latency windows relative to a 100-ms baseline. ANOVAs were used for all statistical tests and were carried out with the Greenhouse-Geisser correction for sphericity departures [72]. ANOVAs were conducted separately for midline and lateral electrodes. Two different analyses were conducted, one taking the different tasks as factor (only correct sentences were included for the morphosyntactic task) and one for the morphosyntactic task alone. ANOVAs for midline electrodes used a repeated-measures design taking as factors sentence Task (semantic or morphosyntactic for the task analysis), Well-/Ill-formed sentences (for the morphosyntactic analysis), Mass/Count, Concreteness (concrete vs. abstract) and Electrodes (Fz, Cz, Pz and Oz). ANOVAs for lateral electrodes also used a repeated-measures design with sentence Task (semantic or morphosyntactic for the task analysis), Well-/Ill-formed sentences (for the morphosyntactic analysis), Mass/Count, Concreteness (concrete vs. abstract), Hemispheres (left vs. right), Localization (three Regions Of Interest [ROIs] or Area; Anterior, Central, and Posterior), and Electrodes (three for each ROI with Anterior including: F3, F7, FC3 and F4, F8, FC4; Central including: C3, Cp1, P3 and C4, Cp2, P4; and Posterior including T3, TP7, O1 and T4, TP8, O2). Results are reported only when significant. In this report, unless otherwise noted, differences were considered significant at p < .05.

### Partial least squares analysis

The Partial Least Square (PLS) analysis is a multivariate data analysis that allows one to identify spatiotemporal relationships between neural activity and experimental design [73], [74]. PLS has the primary advantage, over other multivariate techniques used for EEG analysis (as Principal Component Analysis, PCA, or Independent Component Analysis, ICA), of being able to identify where, simultaneously in space and time, the strongest experimental effects are expressed. PLS is thus able to identify the ERP effects related to the experimental manipulations, and dissociate them from other possible confounding factors.

The term *Partial Least Square* refers to the computation of the optimal least-squares fit to the part of the correlation or the covariance matrix of the data. In this experiment the part is the “cross-block” correlation between the exogenous variables (i.e. the experimental conditions) and the dependent measures (i.e. the ERP amplitude). The ERP input data matrices for the PLS analyses contains subjects and conditions in the rows, and ERP amplitudes for all time points and channels (except for the two ocular electrodes) in the columns. Analysis was restricted to post stimulus interval, from 0 to 700 ms. The input data matrices were first transformed by mean-centering the columns of the ERP data matrix with respect to the grand mean. The averages within each condition were expressed as deviations around zero. The matrix underwent Singular Value Decomposition (SVD) to yield a set of latent variables (LVs). Each of these LV describes how strongly a certain pattern of experimental conditions (design scores) are expressed by each electrode at each time point (electrode salience).

Three outputs were derived from the SVD that serve to interpret the relationships between ERP amplitude and experimental conditions. The first output was a vector of singular values, which represents the un-weighted magnitude of each LV: it is obtained in order to calculate the proportion of the cross-block covariance matrix (i.e., the percentage of task-related variance) attributable to each LV. The second and third outputs contained the structure of the LVs and are orthogonal pairs of vectors (saliences) that are used to identify the temporal and spatial aspects of the latent variables.

The significance of the latent variables singular values was yielded using a permutation test (1000 replications). Permutations consist of sampling without replacement to reassign the order of conditions for each subject. PLS is recalculated for each new permuted sample; the number of times the permuted singular values exceeded the observed singular value in each LV is calculated as a probability. A LV was considered significant at p <.05. To prevent the effects of possible outliers, the stability of the ERP salience in space and time was established through bootstrap re-sampling (200 replications) which provides a standard error. Bootstrap ratios greater than 2.5 were chosen as the cut-off for stable non-zero salience. The main purpose of the bootstrap procedure is to identify those portions of the ERP components that express robust experimental effects across subjects. The Matlab code allowing to perform a PLS analyses can be downloaded at (http://www.rotman-baycrest.on.ca/pls).

## Supporting Information

Appendix S1 **List of experimental stimuli.**
(DOC)
