# Supplementary material for: The Syntactic and Semantic Processing of Mass and Count Nouns: An ERP Study
Source: PLoS One. 2011 Oct 5;6(10):e25885. doi: 10.1371/journal.pone.0025885 (PMC3187832; doi:10.1371/journal.pone.0025885)
Supplement: Appendix S1 — List of experimental stimuli. (DOC) [file pone.0025885.s001.doc]

| **Condition Type** | **Stimulus** | **Translation** |
| --- | --- | --- |
| Count Concrete | pipa | *pipe* |
| Count Concrete | borsa | *bag* |
| Count Concrete | libro | *book* |
| Count Concrete | torta | *cake* |
| Count Concrete | divano | *couch* |
| Count Concrete | fiocco | *bow* |
| Count Concrete | giacca | *jacket* |
| Count Concrete | grotta | *cave* |
| Count Concrete | mulino | *windmill* |
| Count Concrete | sirena | *siren* |
| Count Concrete | tavola | *table* |
| Count Concrete | tromba | *trumpet* |
| Count Concrete | braccio | *arm* |
| Count Concrete | candela | *candle* |
| Count Concrete | capello | *hair* |
| Count Concrete | cavallo | *horse* |
| Count Concrete | fagotto | *bundle* |
| Count Concrete | gambero | *prawn* |
| Count Concrete | gradino | *step* |
| Count Concrete | pisello | *pea* |
| Count Concrete | finestra | *window* |
| Count Concrete | gomitolo | *ball of wool* |
| Count Concrete | impronta | *footprint* |
| Count Concrete | medaglia | *medal* |
| Count Concrete | scodella | *bowl* |
| Count Concrete | tulipano | *tulip* |
| Count Concrete | labirinto | *labyrinth* |
| Count Concrete | scorpione | *scorpion* |
| Count Concrete | guinzaglio | *leash* |
| Count Concrete | pappagallo | *parrot* |
| Count Abstract | lode | *praise* |
| Count Abstract | causa | *cause* |
| Count Abstract | furto | *theft* |
| Count Abstract | prova | *proof* |
| Count Abstract | dramma | *drama* |
| Count Abstract | elenco | *list* |
| Count Abstract | favore | *favour* |
| Count Abstract | legame | *relationship* |
| Count Abstract | novità | *novelty* |
| Count Abstract | pregio | *quality* |
| Count Abstract | rapina | *robbery* |
| Count Abstract | teoria | *theory* |
| Count Abstract | calcolo | *calculation* |
| Count Abstract | delizia | *delight* |
| Count Abstract | difetto | *defect* |
| Count Abstract | insulto | *insult* |
| Count Abstract | ipotesi | *hypothesis* |
| Count Abstract | pretesa | *claim* |
| Count Abstract | sopruso | *abuse (of power)* |
| Count Abstract | commento | *comment* |
| Count Abstract | garanzia | *guarantee* |
| Count Abstract | massacro | *massacre* |
| Count Abstract | principio | *principle* |
| Count Abstract | scandalo | *scandal* |
| Count Abstract | sciagura | *disaster* |
| Count Abstract | sventura | *misfortune* |
| Count Abstract | inchiesta | *investigation* |
| Count Abstract | occasione | *opportunity* |
| Count Abstract | preghiera | *prayer* |
| Count Abstract | scappatoia | *way out* |
| Mass Abstract | fama | *fame* |
| Mass Abstract | calma | *calm* |
| Mass Abstract | garbo | *courtesy* |
| Mass Abstract | pietà | *pity* |
| Mass Abstract | carità | *charity* |
| Mass Abstract | fretta | *hurry* |
| Mass Abstract | igiene | *hygiene* |
| Mass Abstract | lealtà | *loyalty* |
| Mass Abstract | onestà | *honesty* |
| Mass Abstract | quiete | *quiet* |
| Mass Abstract | umiltà | *humility* |
| Mass Abstract | vigore | *vigour* |
| Mass Abstract | chiasso | *din(racket)* |
| Mass Abstract | dignità | *dignity* |
| Mass Abstract | fascino | *charm* |
| Mass Abstract | fiducia | *confidence* |
| Mass Abstract | purezza | *purity* |
| Mass Abstract | terrore | *terror* |
| Mass Abstract | coraggio | *courage* |
| Mass Abstract | costanza | *perseverance* |
| Mass Abstract | pigrizia | *laziness* |
| Mass Abstract | prudenza | *caution* |
| Mass Abstract | rapidità | *speed* |
| Mass Abstract | serenità | *serenity* |
| Mass Abstract | superbia | *haughtiness* |
| Mass Abstract | chiarezza | *clarity* |
| Mass Abstract | disprezzo | *contempt* |
| Mass Abstract | prestigio | *prestige* |
| Mass Abstract | sincerità | *sincerity* |
| Mass Abstract | semplicità | *simplicity* |
| Mass Concrete | lana | *wool* |
| Mass Concrete | burro | *butter* |
| Mass Concrete | calce | *lime* |
| Mass Concrete | grano | *grain* |
| Mass Concrete | latte | *milk* |
| Mass Concrete | canapa | *hemp* |
| Mass Concrete | cipria | *(face) powder* |
| Mass Concrete | cotone | *cotton* |
| Mass Concrete | farina | *flour* |
| Mass Concrete | letame | *manure* |
| Mass Concrete | metano | *methane* |
| Mass Concrete | paglia | *straw* |
| Mass Concrete | piombo | *lead* |
| Mass Concrete | saliva | *saliva* |
| Mass Concrete | sangue | *blood* |
| Mass Concrete | acciaio | *steel* |
| Mass Concrete | baccalà | *stockfish* |
| Mass Concrete | benzina | *petrol* |
| Mass Concrete | canfora | *camphor* |
| Mass Concrete | carbone | *coal* |
| Mass Concrete | gasolio | *diesel fuel* |
| Mass Concrete | muschio | *moss* |
| Mass Concrete | ricotta | *ricotta* |
| Mass Concrete | rugiada | *dew* |
| Mass Concrete | sughero | *cork* |
| Mass Concrete | frumento | *wheat* |
| Mass Concrete | petrolio | *petroleum* |
| Mass Concrete | plastica | *plastic* |
| Mass Concrete | rosmarino | *rosemary* |
| Mass Concrete | inchiostro | *ink* |
